# Genome-Wide Association Study of Smoking Behavior Traits in a Chinese Han Population

**DOI:** 10.3389/fpsyt.2020.564239

**Published:** 2020-09-09

**Authors:** Meng Li, Ying Chen, Jianhua Yao, Sheming Lu, Ying Guan, Yuqiong Xu, Qiang Liu, Silong Sun, Qili Mi, Junpu Mei, Xuemei Li, Mingming Miao, Shancen Zhao, Zhouhai Zhu

**Affiliations:** ^1^ Joint Institute of Tobacco and Health, Yunnan Academy of Tobacco Science, Kunming, China; ^2^ Hangzhou Global Biotechnology and Bioinformatics Co. Ltd, Hangzhou, China

**Keywords:** CUB and sushi multiple domains 1 gene, Han Chinese, immune system, raftlin lipid linker 1 gene, whole-genome sequencing

## Abstract

Tobacco use is one of the leading causes of preventable disease worldwide. Genetic studies have elucidated numerous smoking-associated risk loci in American and European populations. However, genetic determinants for cigarette smoking in Chinese populations are under investigated. In this study, a whole-genome sequencing (WGS)-based genome-wide association study (GWAS) was performed in a Chinese Han population comprising 620 smokers and 564 nonsmokers. Thirteen single-nucleotide polymorphisms (SNPs) of the raftlin lipid linker 1 (RFTN1) gene achieved genome-wide significance levels (P < 5 x 10^−8^) for smoking initiation. The rs139753473 from *RFTN1* and six other suggestively significant loci from CUB and sushi multiple domains 1 (CSMD1) gene were also associated with cigarettes per day (CPD) in an independent Chinese sample consisting of 1,329 subjects (805 smokers and 524 nonsmokers). When treating males separately, associations between smoking initiation and *PCAT5*/*ANKRD30A*, two genes involved in cancer development, were identified and replicated. Within *RFTN1*, two haplotypes (i.e., C-A-C-G and A-G-T-C) formed by rs796812630-rs796584733-rs796349027-rs879511366 and three haplotypes (i.e., T-T-C-C-C, T-T-A-T-T, and C-A-A-T-T) formed by rs879401109-rs879453873-rs75180423-rs541378415-rs796757175 were strongly associated with smoking initiation. In addition, we also revealed two haplotypes (i.e., C-A-G-G and T-C-T-T derived from rs4875371-rs4875372-rs17070935-rs11991366) in the *CSMD1* gene showing a significant association with smoking initiation. Further bioinformatics functional assessment suggested that *RFTN1* may participate in smoking behavior through modulating immune responses or interactions with the glucocorticoid receptor alpha and the androgen receptor. Together, our results may help understand the mechanisms underlying smoking behavior in the Chinese Han population.

## Introduction

Although many programs and regulatory policies of tobacco control have been introduced, reducing the smoking prevalence to a satisfactory level remains an unsolved issue in many countries, especially low- and middle-income countries (1). It has been reported that over 1.3 billion people were tobacco users in 2018 (2). Cigarette smoking is believed to have a wide range of deleterious health effects, such as cardiovascular and pulmonary diseases, and cancers (3–6). Tobacco smoking and second-hand smoke exposure contribute to more than 6 million deaths worldwide annually, posing a serious threat to public health (7).

The establishment of daily smoking usually consists of three main stages: smoking initiation, transition from experimentation to regular smoking, and development of nicotine dependence (ND) (8, 9). Both genetic and environmental factors have been shown to influence all smoking-related stages (9). Smoking initiation, smoking quantity, smoking cessation, and nicotine dependence are commonly studied phenotypes in researches of smoking-related genetic predispositions. Of these, the most well-known is the association between ND and genetics. As primary targets in the brain for nicotine to exert its biological effects, genes encoding nicotinic acetylcholine receptors (nAChRs) represent one of the most investigated ND susceptibility gene families. It is known that nAChRs can trigger the release of dopamine and glutamate, and furthermore reinforce nicotine reward and addiction (10). Meta-analyses and genome-wide association studies (GWASs) have identified a variety of polymorphisms within nAChRs, e.g., rs3743075 in *CHRNA3* (11, 12) and rs2273500 in *CHRNA4* (13) associated with ND. In addition to nAChRs, nicotine metabolizing enzymes (e.g., *CYP2A6*), dopamine receptors and transporters (e.g., *DRD2, DRD4*, and *SLC6A3*), and neuregulin signaling pathway proteins (e.g., *NRG3*) are also considered to have high impacts on nicotine addiction (14–17).

China is the world’s largest producer of tobacco products, and smoking prevalence in Chinese males is among the highest in the world (18, 19). Nevertheless, GWA studies of smoking behaviors in the Han Chinese are much less reported compared with those conducted in the populations of European descent (20) and of European American or of African American populations (21, 22). In the present study, 1,184 Chinese Han adults (including 620 smokers and 564 nonsmokers) were recruited. Whole-genome sequencing (WGS) was performed to identify genome-wide variants. Genetic variants associated with smoking initiation and cigarettes per day (CPD) were determined by association tests. To verify our GWAS findings, a replication analysis was conducted in 1,329 subjects including 805 smokers and 524 nonsmokers. The possible mechanisms of how the observed variants involved in smoking behavior were also briefly discussed.

## Materials and Methods

### Subjects and Phenotypes

The discovery sample included a total of 1,184 unrelated Han Chinese adults from a Yunnan cigarette factory. All participants consented to participate in this project and provided a self-administered survey questionnaire including smoking status, smoking quantity, disease history, height, weight, and age (**Supplementary Table 1**). Among them, 620 were current smokers, 63 were former smokers, and 501 were never smokers. All smokers had smoked at least 100 cigarettes in their lifetimes. The most reported disease among the 1,184 participants was hypertension (32 cases, **Supplementary Table 1**). The study was approved by the institutional review board on human studies.

### Sequencing and Genotyping

For each participant, 5 ml of peripheral blood was collected. DNA was extracted using a QIAamp DNA Mini Kit (Qiagen) according to the manufacturer’s recommendation. Approximately 2 μg of genomic DNA (determined by Qubit Fluorometer, Invitrogen) was prepared for DNA library construction. WGS was performed using the BGISEQ-500 platform (average depth >=30X). Quality control of raw sequences (FASTQ file reads) was conducted by FastQC (v.0.11.7, https://www.bioinformatics.babraham.ac.uk/projects/fastqc/). Clean paired-end reads were mapped against the human reference genome (GRCh37/hg19) by BWA (v.0.7.15) (23). Variants, including SNPs and insertion/deletion (indel) polymorphisms, were called by Genome Analysis Toolkit (GATK, v.3.8, https://gatk.broadinstitute.org/hc/en-us). The discovered SNPs/indels were annotated in the National Center for Biotechnology Information (NCBI) dbSNP database (https://www.ncbi.nlm.nih.gov/snp/).

### Genome-Wide Association Study Quality Control

We applied the following exclusion criteria to filter the samples: 1) mean sequencing depth <10X; 2) 10X coverage <90%; 3) GC content outliers; 4) relative duplication; 5) absolute inbreeding coefficient >1; 6) principal components analysis (PCA) outliers; and 7) sex mismatch.

Quality control of variants was applied by the standard recommended GATK filters, including variant quality score recalibration (VQSR), largest contiguous homopolymer run of the variant allele (HomopolymerRun), binomial test (GetHetCoverage), root mean square of mapping quality (RMSMappingQuality), and strand bias (FisherStrand). To further reduce bias, the following exclusion criteria were adopted: 1) minor allele average depth <4X; 2) average depth in case or control <8; 3) eightfold rate for case or control <0.9; 4) and P-value of Hardy-Weinberg equilibrium test <10^−4^. In addition, variants without dbSNP IDs [also with minor allele frequency (MAF) <0.005] were excluded.

### Statistical Analysis

Individual SNP-based association tests were performed by PLINK (v.1.9) using a logistic regression model (24). Adjusted covariates for association analysis included age, PC1, PC2, and PC3. Manhattan plots were generated using qqman implemented in R (25). The distribution of observed P-values was plotted against that of expected P-values to create a quantile-quantile (QQ) plot through snpStats (v.1.36.0). Haploview software (v.4.2) was employed to determine pairwise linkage disequilibrium (LD) and haplotype blocks (26, 27). Haplotype-based association analyses were examined with the Haplo Stats (v.1.7.9) package (28).

### Replication Analysis

To replicate the GWAS associations, 1,329 participants including 805 smokers and 524 nonsmokers were recruited from local hospitals in Jincheng and Taiyuan of Shanxi Province in China during 2012–2013 (12). All 1,329 participants were males, aged 19–62 years (**Supplementary Table 1**). Participants with psychiatric diseases such as schizophrenia, Alzheimer’s disease, and major depression diagnosed by the Diagnostic and Statistical Manual of Mental Disorders (DSM)-IV criteria were excluded from enrollment. The project was approved by the Ethics Committee of First Affiliated Hospital of Zhejiang University School of Medicine. A set of answers to questions including age, education, income, medical history, environment, and smoking-related behaviors were collected by trained researchers. The sequencing was performed using Illumina HiSeq X10 and analyzed as reported previously (12).

## Results

### Sequencing and Variant Discovery

WGS was carried out in the 1,184 subjects, yielding approximately 1.3 trillion clean reads with an average read length of 100 bp. In the quality control steps for the study participants, as displayed in **Supplementary Table 2**, 26 subjects whose mean sequencing depth was <8X coverage and 37 subjects whose 10X coverage was <80% were removed. The estimate of the inbreeding coefficient and principal component analysis (PCA) filtered out 21 and 3 outliers, respectively.

**Figure 1:**
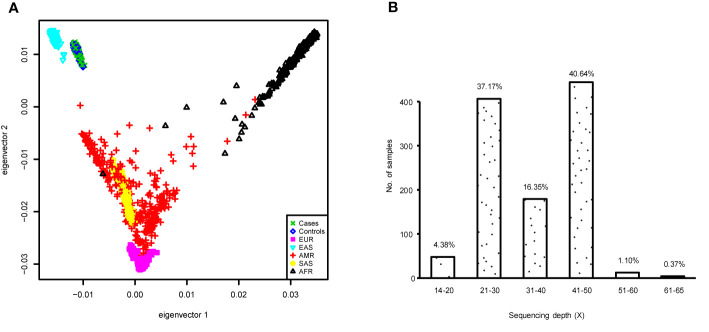
**(A)** Principal component analysis (PCA) plot of subjects enrolled in this study based on two first eigenvectors, built using all cases and controls in the genome-wide association study (GWAS) and, 2,504 individuals of European (CEU), African (YRU), American (AMR), South Asian (SAS), and East Asian (EAS) retrieved from the 1000 Genomes Project phase 3 release. **(B)** Distribution of the sequencing depth for the samples used in the GWAS.

To better demonstrate the population structure of our subjects, we performed PCA on individuals from our study along with samples from European (CEU), African (YRU), American (AMR), South Asian (SAS), and East Asian (EAS) obtained from the phase 3 release of 1000 Genomes Project (29). The distribution of the subjects in this study was concordant with the East Asian cluster (**Figure 1A**). During the test of relatedness, 49 samples were excluded from the analyses because duplicates and cryptic relatedness were detected. A total of 126 samples were removed (data for four subjects were marked as low quality by more than one rule) (**Supplementary Table 2**). In the remaining 1,058 subjects, there were 573 current smokers, 44 ex-smokers, and 441 never smokers (**Table 1**). Smoking prevalence was much higher among men (ever smokers, 50.8%) compared with women (ever smokers, 7.6%). The average sequencing depth was 34.78-fold coverage (**Figure 1B**).

**Table 1:** Characteristics of the discovery sample.

Smoking status	Men (%)	Women (%)	Total (%)
Never	111 (10.5)	330 (31.2)	441 (41.7)
Former	38 (3.6)	6 (0.6)	44 (4.2)
Current	499 (47.2)	74 (7.0)	573 (54.2)

This sample included the 1,058 subjects remaining after quality control steps.

After mapping all clean reads against the human hg19 reference genome, we identified a total of 46,835,008 raw variants (41,383,528 SNPs and 5,451,480 indels). Of them, 7,985,057 SNPs (19.3%) and 3,660,574 indels (67.1%) were eliminated in the variant quality control steps. Finally, 35,189,377 variants remained and were used for genome-wide association analysis (**Supplementary Table 3**). Among them, 1,882,293 were of low frequency (1% < MAF < 5%), and 26,578,915 were rare (MAF < 1%).

### Individual Single-Nucleotide Polymorphism-Based Association Analysis

**Figure 2:**
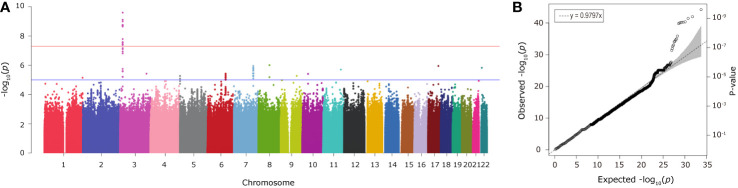
The Manhattan **(A)** and QQ **(B)** plots from association analysis of smoking initiation (ever *vs*. never smokers, with men and women combined). Approximately 12 million variants were tested in the Manhattan plot. The blue and red horizontal lines on the Manhattan plot indicate the thresholds (1 x 10^−5^ and 5 x 10^−8^, respectively). The dashed line on the QQ plot indicates the genomic inflation factor lambda.

**Figure 3:**
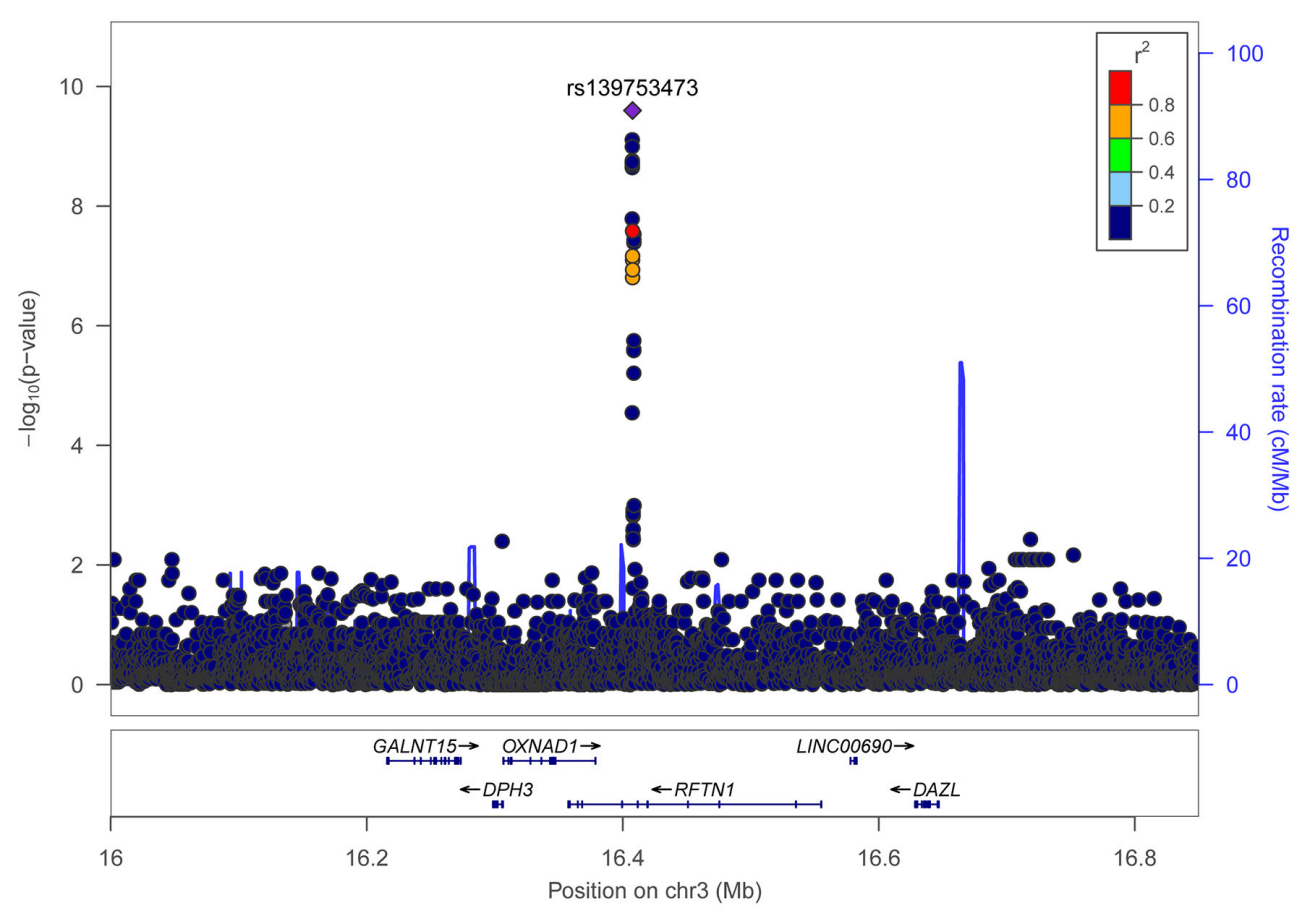
Regional association plot for variants of the RFTN1 gene for smoking initiation. *r* ^2^ values were obtained from the hg19/1000 Genomes Nov 2014 ASN (Han Chinese and Japanese) samples. The plot was generated using LocusZoom (http://locuszoom.sph.umich.edu/).

Individual SNP-based association analysis of smoking initiation was performed with 617 ever smokers *vs.* 441 never smokers (included both males and females). Forty-seven variants displayed statistical significance at a P-value of 10^−5^, including 2 SNPs without a dbSNP ID, **Supplementary Data 1**), 13 of which were considered to be significant at a genome-wide significance level (P < 5 x 10^−8^). Of them, the SNP rs139753473 showed the strongest signal [P = 2.53 x 10^−10^, MAF = 0.0574, odds ratio (OR) = 4.664; **Figure 2** and **Table 2**]. The cluster consisting of rs139753473 and the other 23 SNPs (e.g., rs200713609, rs116358832, and rs796950514) is in close proximity to the Raftlin lipid raft linker 1 (*RFTN1*) gene (**Figure 3**). Of these 24 SNPs, 14 (i.e., rs796139390, rs116358832, rs796931177, rs990470344, rs796687837, rs796950514, rs796881087, rs796068970, rs796689769, rs200713609, rs796257874, rs796606528, rs796468904, and rs796525300, **Supplementary Data 1**) were considered to be low-frequency, i.e., 0.01 < MAF < 0.05. The next top three SNPs were located in pre-mRNA processing factor 31 (*RP11-1102P16.1*, an A/G polymorphism on chromosome 8 position 72324178), phosphatidylinositol transfer protein cytoplasmic 1 (*PITPNC1*, rs190489448), and family with sequence similarity 162 member B (*FAM162B*, rs11153627). In addition, 98 variants from 19 previously reported smoking-associated genes were also identified (P < 10^−3^, **Supplementary Data 1**). For instance, rs140333915 from *HS6ST3* (heparan sulfate 6-O-sulfotransferase 3), rs572691375 from *CYP2C19* (cytochrome P450 family 2 subfamily C member 19), and rs4875371 from *CSMD1* (CUB and sushi multiple domains 1) had P-values <10^−4^. Of these, the rs140333915 and rs572691375 were low-frequency and rare variants (MAF = 0.0146 and 0.0073, respectively). When treating males separately, we found one genome-wide significant signal, rs11010435 (P = 4.72 x 10^−8^) located between *PCAT5* (prostate cancer associated transcript 5) and *ANKRD30A* (ankyrin repeat domain 30A) and 98 variants from 31 known smoking-associated genes achieved P values of 10^−3^ (**Supplementary Data 1**). However, the corresponding QQ plot was not well-behaved (**Supplementary Figure 1A**).

**Table 2:** Top 20 genetic loci associated with smoking initiation.

Gene	SNP ID	Chr	Position	Minor/major allele	MAF	P (Fisher)	OR
*RFTN1*	rs139753473	3	16407626	A/G	0.0574	2.53E−10	4.664
*RFTN1*	rs200713609	3	16407433	A/G	0.0438	7.73E−10	6.240
*RFTN1*	rs116358832	3	16407473	G/A	0.0380	1.01E−09	7.904
*RFTN1*	rs796950514	3	16407447	C/T	0.0425	1.73E−09	6.066
*RFTN1*	rs796881087	3	16407459	G/A	0.0425	1.84E−09	6.052
*RFTN1*	rs796687837	3	16407457	G/A	0.0424	1.90E−09	6.045
*RFTN1*	rs796068970	3	16407449	G/A	0.0425	2.04E−09	6.028
*RFTN1*	rs796689769	3	16407441	T/C	0.0425	2.26E−09	6.003
*RFTN1*	rs796931177	3	16407376	A/T	0.0410	1.63E−08	5.245
*RFTN1*	rs796525300	3	16407640	A/C	0.0477	2.59E−08	4.298
*RFTN1*	rs75180423	3	16408723	A/C	0.0655	2.94E−08	3.239
*RFTN1*	rs796757175	3	16408752	T/C	0.0666	3.68E−08	3.164
*RFTN1*	rs541378415	3	16408740	T/C	0.0665	4.08E−08	3.152
*RFTN1*	rs796257874	3	16407658	A/G	0.0448	6.80E−08	4.306
*RFTN1*	rs796468904	3	16407651	A/T	0.0462	7.91E−08	4.121
*RFTN1*	rs990470344	3	16407683	T/C	0.0421	1.16E−07	4.395
*RFTN1*	rs796606528	3	16407656	G/A	0.0453	1.56E−07	4.014
*RP11-1102P16.1*	8-72324178	8	72324178	A/G	0.2845	9.75E−07	1.633
*PITPNC1*	rs190489448	17	65640344	T/C	0.0128	1.13E−06	0.091
*RFTN1*	rs879511366	3	16408625	C/G	0.0562	1.77E−06	2.914

The term "Fisher" denotes Fisher’s exact test. Chr, chromosome; MAF, minor allele frequency; OR, odds ratio.

Genome-wide association analyses for smoking quantity were conducted among 573 current smokers. With male and female smokers were combined, no genome-wide significant signals were identified (**Supplementary Figure 1B** and **Supplementary Data 1**). Despite this, 45 variants reached a P value of <10^−5^, with the rs78955061 from the intergenic region of *ACKR3* (atypical chemokine receptor 3) and *LOC93463* had the smallest P value of 7.92 x 10^−7^. With a cutoff of p <10^−3^, 27 variants from 19 known smoking-associated genes were determined. When the study sample was restricted to males, 22 SNPs yielded a P value of <10^−5^, of which the rs143124048 from *PALLD* (a gene encoding palladin protein) had the smallest P value of 1.70 x 10^−6^ (**Supplementary Data 1**), and 28 SNPs from 15 known smoking-associated genes were identified at p <10^−3^. However, none of these signals reached a genome-wide significant level, which may be due to a lack of statistical power (**Supplementary Figure 1C**).

### Replication Study

Due to the fact that very few females smoke in China, only males were included in the replication sample. Variants which had a P-value lower than 10^−5^ in the primary analysis were selected (for *RFTN1* and *CSMD1*, all variants with P-value <10^−3^ were tested). As a result, none of the smoking initiation or CPD associated loci from the total discovery sample (with men and women combined) were significant. However, 18 male-specific smoking initiation associated loci (i.e., rs10128115, rs10128145, rs72795203, rs12241402, rs10128497, rs16936694, rs7072685, rs12261634, rs11010478, rs11010482, rs112089093, rs12248963, rs1480525, rs10128398, rs12256178, rs10128169, rs1122458, and rs7071386) yielded significant results (P < 0.05, **Supplementary Table 4**). All of these SNPs were located in the intergenic region between *PCAT5* and *ANKRD30A*. At a P-value threshold of 0.10, another 15 SNPs also showed evidence for replication, 14 of which were in the intergenic region between *PCAT5* and *ANKRD30A*. The other one, rs4590382 (P = 0.06), was an intergenic SNP between *LOC101928283* and *GRM8*. For male-specific CPD- associated loci in the primary analysis, no evidence of replication was observed. Intriguingly, in testing for an association with CPD, the rs139753473 within *RFTN1* and six SNPs within *CSMD1* showed a P-value of <0.05 (**Table 3**). The six SNPs from *CSMD1* included rs76965088, rs117740219, rs78094590, rs138695620, rs76195425, and rs148939406. Furthermore, one SNP from *RFTN1* (rs796139390), and four SNPs from *CSMD1* (i.e., rs114254701, rs10503200, rs56391646, and rs149909271) achieved a nominally marginal significance level (P < 0.10) for an association with CPD.

**Table 3:** Cigarettes per day (CPD)-associated single-nucleotide polymorphisms (SNPs) within *RFTN1* and *CSMD1* in the replication sample.

Gene	SNP ID	Minor/major allele	MAF	P^*^	Beta
*RFTN1*	rs139753473	A/G	0.0685	**0.0460**	−1.263
*RFTN1*	rs796139390	C/G	0.0903	0.0600	−1.068
*CSMD1*	rs76965088	T/C	0.0267	**0.0024**	2.809
*CSMD1*	rs117740219	T/C	0.0248	**0.0160**	2.345
*CSMD1*	rs78094590	T/C	0.0260	**0.0341**	2.063
*CSMD1*	rs138695620	C/A	0.0245	**0.0367**	2.108
*CSMD1*	rs76195425	A/G	0.0252	**0.0431**	2.018
*CSMD1*	rs148939406	T/C	0.0230	**0.0449**	2.135
*CSMD1*	rs114254701	A/T	0.0241	0.0572	1.938
*CSMD1*	rs10503200	G/A	0.0267	0.0630	1.706
*CSMD1*	rs56391646	A/T	0.0241	0.0781	1.803
*CSMD1*	rs149909271	G/C	0.0196	0.0918	1.939

*Nominally significant association (i.e., P < 0.05) is shown in boldface type.

### Haplotype-Based Association Analysis of *RFTN1* and *CSMD1*

**Figure 4:**
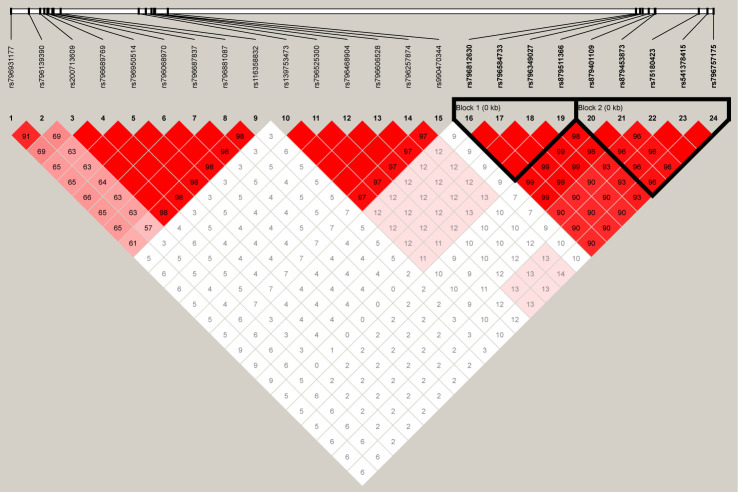
The linkage disequilibrium (LD) plot depicts the structure of haplotype blocks encompassing the 24 single-nucleotide polymorphisms (SNPs) located in the RFTN1 gene in our Chinese Han population. This plot was generated using Haploview (https://www.broadinstitute.org/haploview/haploview). The standard Haploview LD color scheme based on D’ has been applied. The value of 100 x D’ is displayed for each SNP pair’s tile unless D’ = 1.

For the 24 SNPs in *RFTN1* with a P-value of <10^−3^ according to the results from individual SNP-based association analysis for smoking initiation, two LD blocks were identified (D’ > 0.97) (**Figure 4**). Association tests between these haplotypes and smoking initiation revealed that two haplotypes, C-A-C-G and A-G-T-C, formed by rs796812630, rs796584733, rs796349027, and rs879511366 were significantly associated with smoking initiation under the additive model (Hap-Score = −4.57 and 4.63, P = 4.83 x 10^−6^ and 3.65 x 10^−6^, respectively, **Table 4**). In addition, three haplotypes, T-T-C-C-C, T-T-A-T-T, and C-A-A-T-T, constituted by rs879401109-rs879453873-rs75180423-rs541378415-rs796757175 also correlated with smoking initiation (Hap-Score = −5.33, 3.10 and 4.50, P = 1.00 x 10^−7^, 1.94 x 10^−3^ and 6.91 x 10^−6^, respectively, **Table 4**).

**Table 4:** Haplotypes of *RFTN1* and *CSMD1* genes associated with smoking initiation.

Gene	SNP combination	Haplotype	Hap-Freq	Hap-Score	p-Hap*	p-Global
*RFTN1*	rs796812630-rs796584733-rs796349027-rs879511366	C-A-C-G	0.94	−4.57	**4.83E-06**	2.15E−05
		A-G-T-G	0.00	0.10	0.92	
		A-G-T-C	0.06	4.63	**3.65E-06**	
	rs879401109-rs879453873-rs75180423-rs541378415-rs796757175	T-T-C-C-C	0.93	−5.33	**1.00E-07**	6.90E−07
		T-T-A-T-T	0.01	3.10	**1.94E-03**	
		C-A-A-T-T	0.05	4.50	**6.91E-06**	
*CSMD1*	rs4875371-rs4875372-rs17070935-rs11991366	C-A-G-G	0.14	−3.35	**3.36E-04**	4.70E-04
		T-C-G-G	0.01	0.39	0.69	
		T-C-T-T	0.85	3.77	**1.61E-04**	

*Significant association after Bonferroni correction is shown in boldface type. The corrected P value thresholds after Bonferroni test for rs796812630-rs796584733-rs796349027-rs879511366, rs879401109-rs879453873-rs75180423-rs541378415-rs796757175, and rs4875371-rs4875372-rs17070935-rs11991366 were 0.0125 (0.05/4), 0.01 (0.05/5), and 0.0125 (0.05/4), respectively.

Given that in the individual SNP-based association analysis of smoking initiation, the *CSMD1* had the largest number of SNPs with P <10^−3^, of the reported smoking-associated genes, haplotype-based association analysis was also performed on the 29 SNPs in *CSMD1*. One LD block exhibited a D’ larger than 0.97 (**Supplementary Figure 2**). As shown in **Table 4**, two haplotypes, C-A-G-G and T-C-T-T, derived from rs4875371-rs4875372-rs17070935-rs11991366, were strongly associated with smoking initiation (Hap-Score = −3.35 and 3.77, P = 3.36 x 10^-4^ and 1.61 x 10^-4^, respectively).

### Bioinformatics Functional Assessment of *RFTN1*

*In silico* functional analyses based on the RegulomeDB (https://regulomedb.org/) and HaploReg (https://pubs.broadinstitute.org/mammals/haploreg/haploreg.php) databases were performed for SNPs with P <10^−8^, i.e., rs139753473, rs200713609, rs116358832, rs796950514, rs796881087, rs796687837, rs796068970, and rs796689769. All of these SNPs were intron variants within *RFTN1*. The male-specific smoking associated rs11010435 was omitted because the GWAS did not have adequate power and this locus was not replicated. Although evidence of regulatory potential was weak for rs139753473 (RegulomeDB score = 0.008), this SNP alters E2F, Egr-1, MOVO, Nrf1, UF1H3BETA, YY1, and SP1 transcription factor binding motifs according to HaploReg (**Supplementary Table 5**). A further investigation using the PROMO prediction tool (30) suggested that the locus of rs139753473 interacted with two transcription factors, including the glucocorticoid receptor alpha and the androgen receptor. Interestingly, the glucocorticoid receptor has been reported to be associated with the probability of smoking severity and cessation in a sample of obstructive airway disease patients (31). It has been suggested that cigarette smoking could increase androgen receptor activity (32). Additionally, rs200713609 had a RegulomeDB score of 0.61 and could alter the PEBP transcription factor binding motif. The RegulomeDB score for rs116358832 was 0.13. Motifs altered by rs116358832 included CEBPB and GATA. For rs796950514, rs796881087, rs796687837, rs796068970, and rs796689769, the RegulomeDB score ranked from 0.13 to 0.61, and no altered motif was found in either RegulomeDB or HaploReg. In the examination of the correlation between these variants and the expression of *RFTN1*, no available expression quantitative trait loci (eQTL) data could be found. Furthermore, according to the expression pattern retrieved from GTEX PORTAL (https://gtexportal.org/home/), *RFTN1* had the highest expression in lymphocytes and was also expressed in various brain tissues, such as the cortex, the frontal cortex (BA9), and cerebellum (**Supplementary Figure 3**).

## Discussion

Associations between genetic variants and cigarette smoking have been largely deciphered for European- and American-ancestry populations (21, 33). For the Chinese Han population, studies on genetic factors conferring smoking susceptibility are still limited in the literature. Here we performed deep WGS of 1,184 Chinese samples and discovered 35 million variants. Of them, 1,882,293 (5%) and 26,578,915 (76%) were found to be low-frequency and rare variants, respectively. Follow up replication analyses revealed risk alleles in *RFTN1*, *CSMD1*, and *PCAT5*/*ANKRD30A* genes likely contributing to smoking behavior.

In the discover stage, 13 SNPs from *RFTN1* were significantly associated with smoking initiation, i.e., rs139753473, rs200713609, rs116358832, rs796950514, rs796881087, rs796687837, rs796068970, rs796689769, rs796931177, rs796525300, rs75180423, rs796757175, and rs541378415. The rs11010435 from the intergenic region of *PCAT5* and *ANKRD30A* was also significantly associated with smoking initiation in male smokers. For CPD, we found no genome-wide significant signals, but there were 45 and 22 variants in the total sample and the male subgroup, respectively, at the threshold of P <10^−5^. To validate the preliminary findings, we performed a replication study for the variants with a P-value less than 10^−5^ (for *RFTN1* and *CSMD1*, variants with P <10^−3^ were included), using another Chinese Han sample containing 1,329 male subjects. Although variants associated with smoking initiation and CPD in the total discovery sample were not replicated, we replicated 18 loci for their association with smoking initiation in men, which including rs10128115, rs10128145, rs72795203, rs12241402, rs10128497, rs16936694, rs7072685, rs12261634, rs11010478, rs11010482, rs112089093, rs12248963, rs1480525, rs10128398, rs12256178, rs10128169, rs1122458, and rs7071386 from the intergenic region between *PCAT5* and *ANKRD30A* (P < 0.05). For male-specific associations with CPD, no evidence of replication was found. Furthermore, although *RFTN1* and *CSMD1* were originally identified in the test of smoking initiation, in the replication test of CPD, the *RFTN1* gene’s rs139753473 and the *CSMD1* gene’s rs76965088, rs117740219, rs78094590, rs138695620, rs76195425, and rs148939406 reached a P-value of <0.05. Given that smoking initiation and CPD are moderately correlated smoking behavior traits (correlation coefficient r = 0.425, P = 2.6 x 10^−15^) (34), these association results for *RFTN1* and *CSMD1* are of great interest and warranted to elucidate their biological roles in smoking.

Within the 24 SNPs observed in the *RFTN1*, two LD blocks, rs796812630-rs796584733-rs796349027-rs879511366 and rs879401109-rs879453873-rs75180423-rs541378415-rs796757175, were uncovered. Two haplotypes (i.e., C-A-C-G and A-G-T-C) from the former and three (i.e., T-T-C-C-C, T-T-A-T-T, and C-A-A-T-T) from the latter were significantly associated with smoking initiation. Additionally, haplotype-based association analysis also showed that two *CSMD1*-derived haplotypes, C-A-G-G and T-C-T-T formed by rs4875371-rs4875372-rs17070935-rs11991366 were strongly correlated with smoking initiation.

The lead SNP (rs139753473) associated with smoking initiation is located within the intron region of the RFTN1 gene. It may bind to two transcription factors, i.e., the glucocorticoid receptor alpha and the androgen receptor, which have been proposed to play a role in smoking. Nonetheless, the functional studies were carried out *in silico* and need experimental validation. Although *RFTN1* had the highest expression in lymphocytes, its expression can also be found in brain tissues, e.g., the cortex, frontal cortex (BA9), and cerebellum. These regions are believed to be involved in the brain’s reward and inhibitory control processes (35, 36). In addition, *RFTN1* contributes to multiple immune-related biological pathways, including B and T cell receptor (BCR and TCR, respectively) signaling, toll-like receptor (TLR) 3 signaling, and interleukin-17 (IL17) production (37–40). It has been increasingly recognized that activation of central immune signaling by substances (e.g., opioids) can enhance drug reward (41, 42). In mice, TLR3 modulates cocaine reward through pro-inflammatory immune signaling (43). In particular, BCR, TCR, TLR, and IL17 induce activation of nuclear factor kappaB (NF-κB) (44–47), and NF-κB mediates the reward effects of drugs (e.g., cocaine) (48). Moreover, NF-κB is not only a transcription factor involved in inflammation and the immune response (49), but is also a regulator of synaptic plasticity and memory (50).It is plausible that *RFTN1* could play a role in smoking initiation by regulating immune responsiveness.

*CSMD1* is a complement-regulatory protein that is highly expressed in the central nervous system, contributing to addiction vulnerability (51). In an analysis of 4,122 psoriasis cases and 3,101 healthy controls, *CSMD1* showed evidence of association with cigarette smoking (52). Regarding *PCAT5* and *ANKRD30A*, it is reported that both of them are related to cancer progression. For instance, *PCAT5* is a long noncoding RNA regulated by the ERG, an active transcription factor common in human prostate cancer (53). Similarly, the *ANKRD30A* encodes a DNA-binding transcription factor implicated in breast cancer (54, 55). Our findings indicated that these two genes could be potential targets to investigate the connection between smoking and cancers.

One major limitation of this study is the sample size. Due to the relatively high sequencing cost per sample and difficulty in recruiting participants, our sample size was not large enough to provide various phenotypes and adequate statistical power. Although the application of WGS in genome-wide association analysis allowed for successful detection of a few novel or low-frequency variants associated with cigarette smoking, these SNPs are infrequently reported in previous smoking or other psychiatric‐related traits, preventing further functional assessments. This also raised the question of whether the observed loci, especially those from *RFTN1* and *PCAT5/ANKRD30A*, were specifically associated with smoking in the Chinese population. Further studies with particular attention to these genes are therefore required to address this issue. Another concern is that smoking prevalence is usually different by sex, while in this study, association analyses were not performed in a female-specific manner, because the discovery cohorts only included 74 current female smokers and there were no females in the replication sample. In addition, the Fagerström Test for Nicotine Dependence (FTND) is a widely used instrument to estimate nicotine dependence. It is possible that GWAS of this phenotype may produce more robust results. Additionally, as a major metabolite of nicotine, cotinine represents a direct biomarker of smoking quantity. Measuring the cotinine level in serum, urine, or saliva could benefit further validation studies, by providing more objective information on smoking quantity.

In summary, to the best of our knowledge, we have reported the first WGS-based GWAS of smoking phenotypes in a Chinese Han cohort. We provided exploratory evidence that *RFTN1* and *CSMD1* are involved in smoking. Associations between smoking initiation and *PCAT5*/*ANKRD30A* were also detected and replicated in a male-specific manner. *RFTN1* might function in smoking initiation through interactions with the immune system, the glucocorticoid receptor alpha and androgen receptor signaling. These findings provide extensive insight into the biological mechanisms of smoking behavior in the Chinese Han population.

## Data Availability Statement

The datasets presented in this article are not readily available because due to the restriction of “Regulation of the People’s Republic of China on the Administration of Human Genetic Resources”, sequencing data of this study can not be shared publicly. Requests to access the datasets should be directed to zhuzhouhai@gmail.com.

## Ethics Statement

The studies involving human participants were reviewed and approved by Biomedical Ethics Committee of Joint Institute of Tobacco and Health. The patients/participants provided their written informed consent to participate in this study.

## Author Contributions

ML, JY, and ZZ conceived the study. YC, ML, and QL performed the data analysis. ML wrote the manuscript. All authors contributed to the article and approved the submitted version.

## Conflict of Interest

The Joint Institute of Tobacco and Health received research grants from China Tobacco Yunnan Industrial CO. LTD. Author QL was employed by the Hangzhou Global Biotechnology and Bioinformatics CO. LTD. However, this study was carried out independently from either the funding agency or employer.

The remaining authors declare that the research was conducted in the absence of any commercial or financial relationships that could be construed as a potential conflict of interest.

## Supplementary Material

Supplementary data 1 Association analysis results of smoking initiation and CPD in this study.

Supplementary data 2 This file contains **Supplementary Figures 1-3**.

Supplementary data 3 This file contains **Supplementary Tables 1-5**.
