# Supplementary material for: Genome-Wide Association Study of Smoking Behavior Traits in a Chinese Han Population
Source: Front Psychiatry. 2020 Sep 9;11:564239. doi: 10.3389/fpsyt.2020.564239 (PMC7509597; doi:10.3389/fpsyt.2020.564239)
Supplement: Supplementary data 2 — This file contains Supplementary Figures 1-3 . [file DataSheet_2.docx]

# Supplementary figures


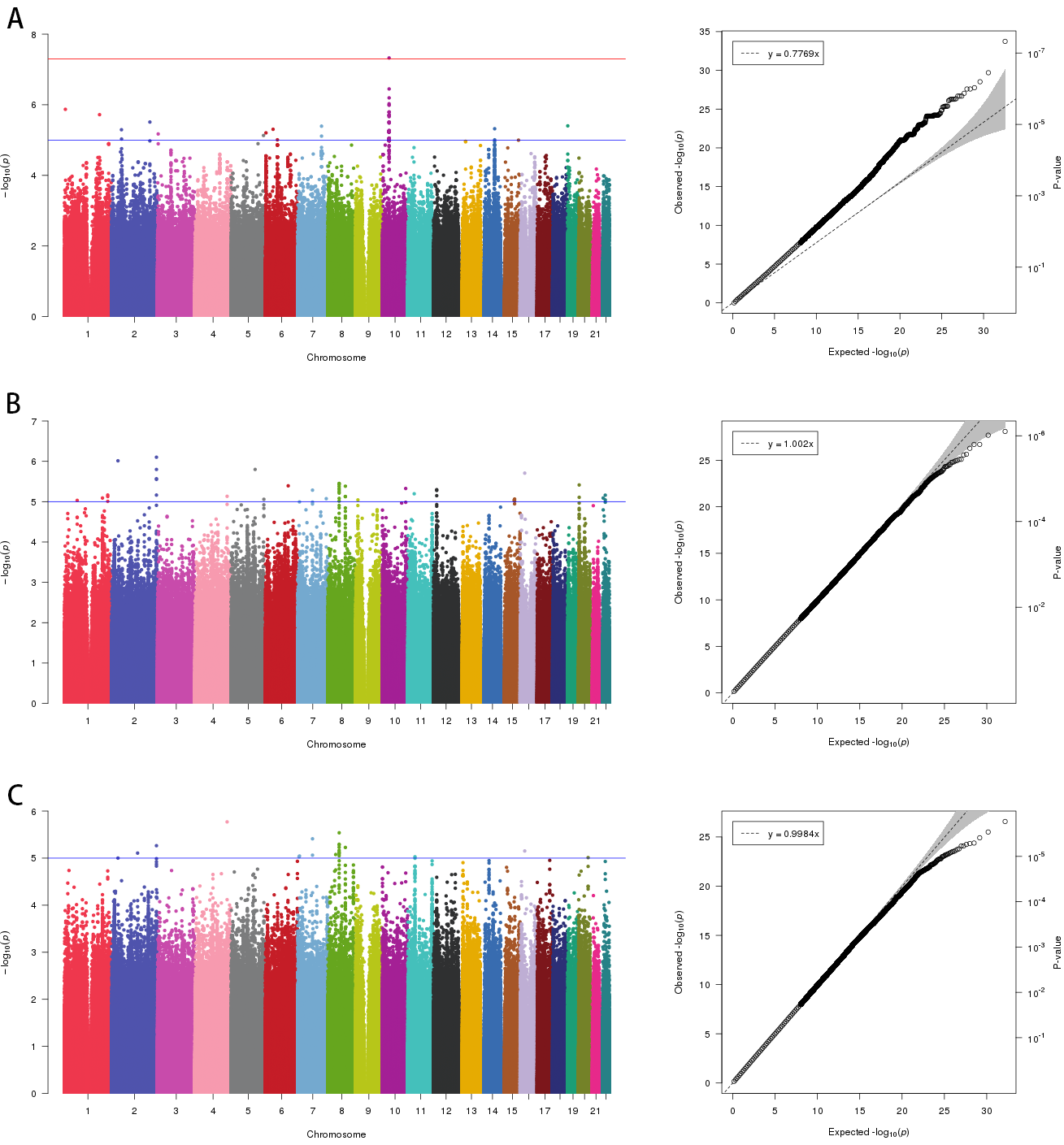


Supplementary figure 1 A: The Manhattan (left) and QQ (right) plots for the association with smoking status (current vs. never smokers) among men. About 12 million variants were tested in this Manhattan plot; B: The Manhattan (left) and QQ (right) plots for the association with CPD (men and women combined). 5 million variants were tested in this Manhattan plot; C: The Manhattan (left) and QQ (right) plots for the association with CPD among men. 5 million variants were tested in this Manhattan plot. The blue and red horizontal lines on the Manhattan plot indicate the thresholds (1 x 10^−5^ and 5 x 10^-8^, respectively). The dashed line on the QQ plot indicates the genomic inflation factor lambda.


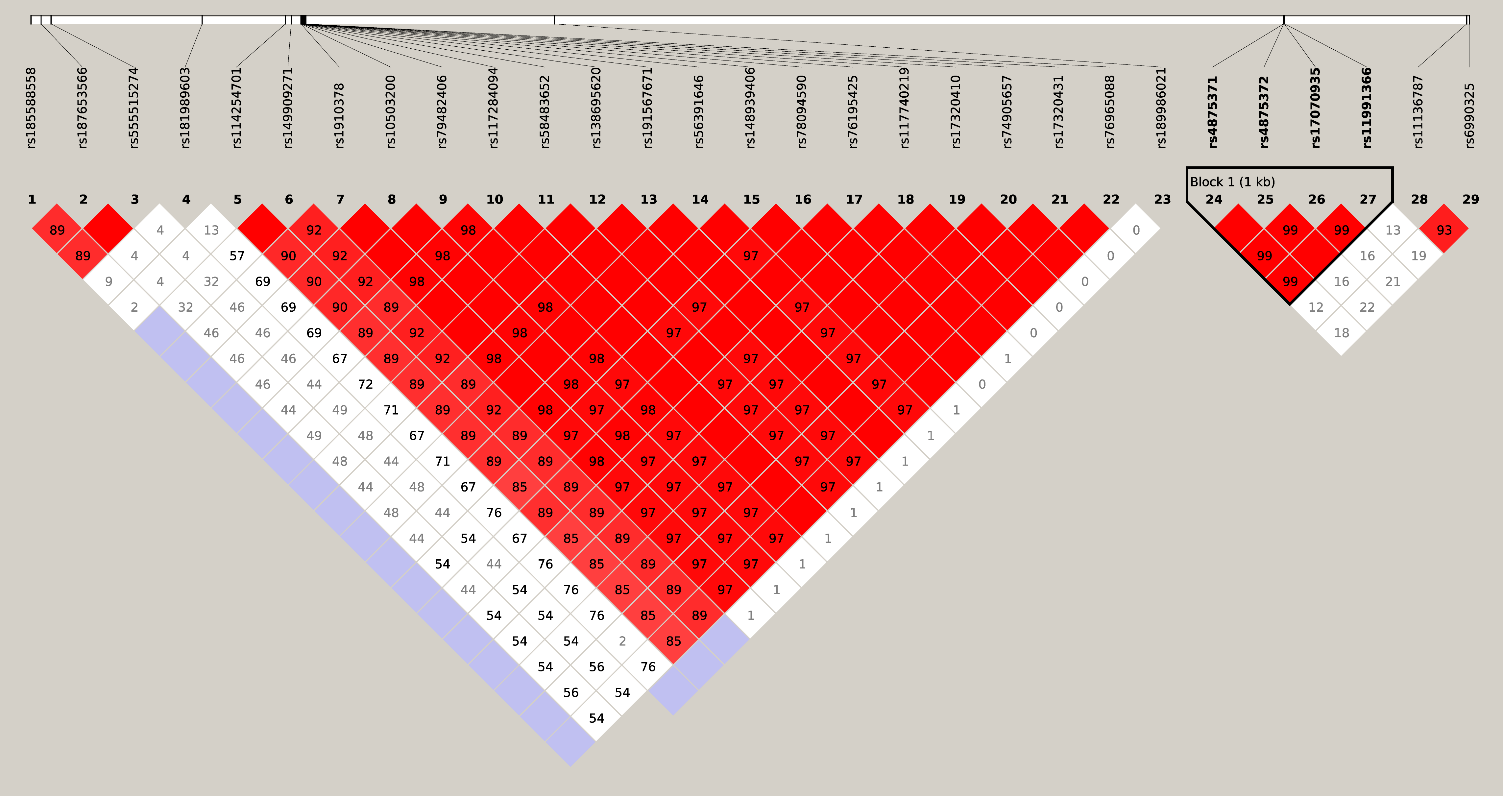


Supplementary figure 2 LD structure for SNPs on CSMD1 gene in our Chinese Han samples.


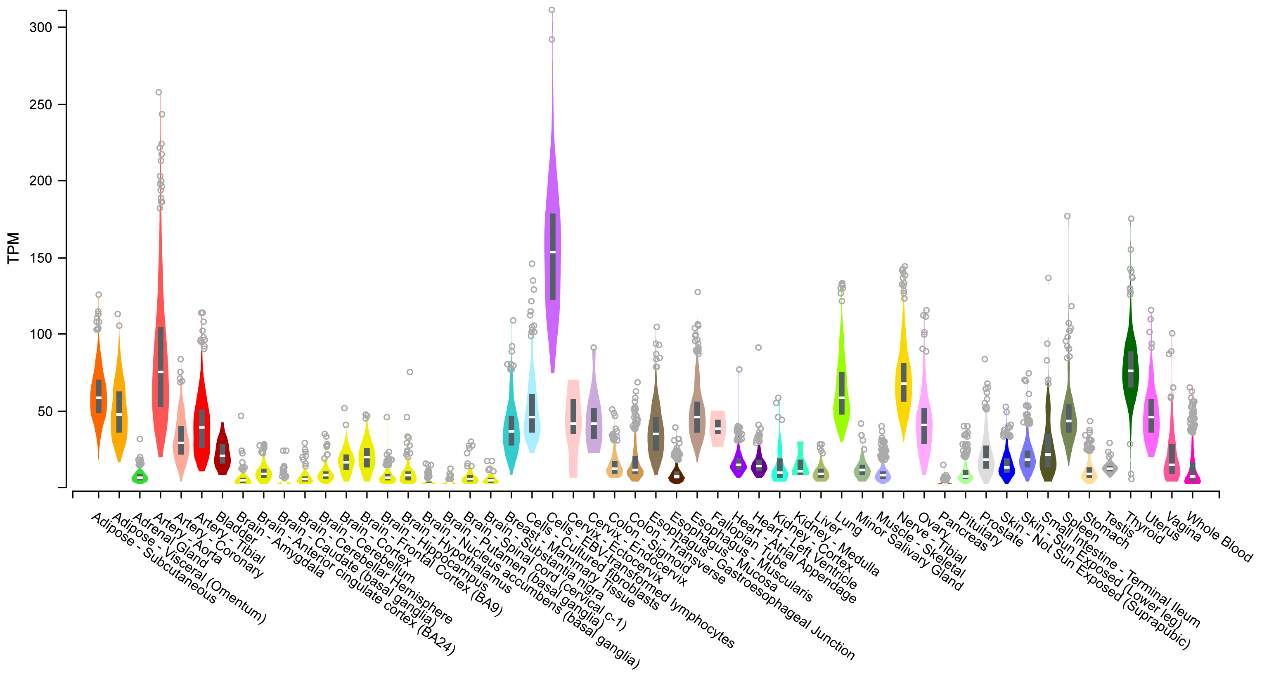


Supplementary figure 3 Expression profile of RFTN1 gene obtained from The Genotype-Tissue Expression (GTEx) project website (https://www.gtexportal.org/home/).
