# Supplementary material for: Genome-Wide Association Study of Smoking Behavior Traits in a Chinese Han Population
Source: Front Psychiatry. 2020 Sep 9;11:564239. doi: 10.3389/fpsyt.2020.564239 (PMC7509597; doi:10.3389/fpsyt.2020.564239)
Supplement: Supplementary data 3 — This file contains Supplementary Tables 1-5 . [file DataSheet_3.docx]

**Supplementary table 1 Demographic characteristics of the participants.**

|  | **Current smoker** | | | **Former smoker** | | | | **Never smoker** | | |
| --- | --- | --- | --- | --- | --- | --- | --- | --- | --- | --- |
|  | **Male** | | **Female** | **Male** | | **Female** | | **Male** | | **Female** |
| Discovery sample | | | | | | | | | | |
| Age | | | | | | | | | | |
| 20-30 | 28 | | 16 | 1 | | 0 | | 10 | | 78 |
| 31-40 | 96 | | 12 | 4 | | 0 | | 30 | | 97 |
| 41-50 | 263 | | 23 | 13 | | 2 | | 49 | | 80 |
| 51-60 | 112 | | 23 | 20 | | 4 | | 22 | | 75 |
| Height (cm) | | | | | | | | | | |
| <=160 | 27 | | 15 | 2 | | 2 | | 2 | | 16 |
| 161-170 | 208 | | 56 | 17 | | 2 | | 51 | | 276 |
| 171-180 | 230 | | 3 | 16 | | 2 | | 55 | | 36 |
| >=181 | 34 | | 0 | 3 | | 0 | | 3 | | 2 |
| Weight (kg) | | | | | | | | | | |
| <=45 | 0 | | 5 | 0 | | 2 | | 0 | | 30 |
| 46-55 | 33 | | 12 | 9 | | 1 | | 0 | | 79 |
| 56-65 | 125 | | 16 | 1 | | 1 | | 16 | | 82 |
| 66-75 | 223 | | 21 | 9 | | 2 | | 16 | | 76 |
| >=76 | 118 | | 20 | 19 | | 0 | | 79 | | 63 |
| Replication sample | | | | | | | | | | |
| Age | | | | | | | | | | |
| 19-30 | 156 | |  |  | |  | | 61 | |  |
| 31-40 | 222 | |  |  | |  | | 177 | |  |
| 41-50 | 314 | |  |  | |  | | 239 | |  |
| 51-62 | 113 | |  |  | |  | | 47 | |  |
| Height (cm) | | | | | | | | | | |
| <=160 | 22 | |  |  | |  | | 37 | |  |
| 161-170 | 388 | |  |  | |  | | 273 | |  |
| 171-180 | 372 | |  |  | |  | | 203 | |  |
| >=181 | 23 | |  |  | |  | | 10 | |  |
| Weight (kg) | | | | | | | | | | |
| 46-55 | 49 | |  |  | |  | | 15 | |  |
| 56-65 | 207 | |  |  | |  | | 117 | |  |
| 66-75 | 318 | |  |  | |  | | 232 | |  |
| >=76 | 231 | |  |  | |  | | 158 | |  |
| Top 15 Reported diseases reported in Discovery sample | | | | | | | | | | |
| Disease | | Discovery sample | | | | | Replication sample | | | |
|  |  | Smoker | | | Non-smoker | | Smoker | | Non-smoker | |
| Hypertension | | 18 | | | 14 | | 228 | | 122 | |
| Lithiasis | | 10 | | | 10 | | NA | | NA | |
| Diabetes | | 10 | | | 4 | | 1 | | 1 | |
| Uarthritis | | 7 | | | 3 | | NA | | NA | |
| Cancer | | 6 | | | 7 | | NA | | NA | |
| Hyperthyroidism | | 5 | | | 2 | | NA | | NA | |
| Enterogastritis | | 4 | | | 3 | | NA | | NA | |
| Bronchitis | | 4 | | | 2 | | NA | | NA | |
| Rhinitis | | 3 | | | 3 | | NA | | NA | |
| Hyperlipaemia | | 3 | | | 1 | | NA | | NA | |
| Cholecystitis | | 2 | | | 2 | | NA | | NA | |
| Steatohepatitis | | 3 | | | 1 | | NA | | NA | |
| Chronic Pharyngitis | | 3 | | | 1 | | NA | | NA | |
| Pneumonia | | 3 | | | 0 | | NA | | NA | |
| Pulmonary Tuberculosis | | 2 | | | 1 | | NA | | NA | |

Note: In this table, the discovery sample included the 1058 subjects remained after quality control steps. The replication sample included the 1329 subjects used in the replication study. Reported disease was counted regardless sex. A subject might report more than one disease.

**Supplementary table 2 Samples filtered during quality control.**

| **Filter** | **Criteria** | **Removed^*^** |
| --- | --- | --- |
| Mean sequencing depth | Fold coverage < 8X | 26 |
| Coverage | 10X coverage < 80% | 37 |
| GC content | Outliers | 0 |
| Relatedness | Duplication/1^st^-degree/2^nd^-degree | 49 |
| Inbreeding coefficient | Outliers | 21 |
| PCA | Outliers | 3 |
| Total | - | 126 |

* 4 subjects were filtered by more than one rules.

**Supplementary table 3 Variants filtered during quality control.**

| Filter | Criteria | SNP Removed | InDel Removed |
| --- | --- | --- | --- |
| Depth for minor allele | Minor allele average depth < 4 | 1,513,454 | 641,197 |
| Depth for site | Case or control average depth < 8 | 550,211 | 249,950 |
| VQSR | Filtered | 1,602,533 | 1,068,993 |
| 8X depth fraction of site | Case or control 8X rate < 0.9 | 2,340,329 | 1,115,249 |
| Homopolymer | Homopolymer run>=6 | 5,602,776 | 2,773,445 |
| Allelic balance in heterozygotes | Binomial test P-value < 1.0E-4 and bias>0.5 | 2,327,551 | NA |
| Mapping quality score | Root mean square <30 | 1,048,057 | 403,590 |
| Strand bias | Fisher test P-value < 1.0E-5 and OR > 3 | 956,266 | NA |
| Hardy-Weinberg disequilibrium | P < 1.0E-4 for control samples | 139,902 | |

**Supplementary table 4 Male-specific signals associated with smoking initiation detected in the replication sample.**

| **Gene** | **SNP ID** | **Chromosome** | **Position** | **Minor allele frequency** | **Minor / major allele** | **P** | |
| --- | --- | --- | --- | --- | --- | --- | --- |
|  |  |  |  |  |  | **Discovery stage** | **Replication stage** |
| PCAT5,ANKRD30A | rs10128115 | 10 | 36414646 | 0.2201 | C/T | 5.88E-06 | 0.01233 |
| PCAT5,ANKRD30A | rs10128145 | 10 | 36412261 | 0.2201 | C/A | 5.88E-06 | 0.01417 |
| PCAT5,ANKRD30A | rs72795203 | 10 | 36415292 | 0.2209 | G/A | 8.94E-06 | 0.01722 |
| PCAT5,ANKRD30A | rs12241402 | 10 | 36423465 | 0.2193 | T/C | 5.53E-06 | 0.01837 |
| PCAT5,ANKRD30A | rs10128497 | 10 | 36412327 | 0.2209 | C/T | 8.94E-06 | 0.01958 |
| PCAT5,ANKRD30A | rs16936694 | 10 | 36420856 | 0.2193 | T/G | 5.53E-06 | 0.0215 |
| PCAT5,ANKRD30A | rs7072685 | 10 | 36407372 | 0.2186 | A/G | 5.53E-06 | 0.02775 |
| PCAT5,ANKRD30A | rs12261634 | 10 | 36401285 | 0.2209 | G/A | 8.94E-06 | 0.02986 |
| PCAT5,ANKRD30A | rs11010478 | 10 | 36413108 | 0.2193 | T/G | 5.53E-06 | 0.03587 |
| PCAT5,ANKRD30A | rs11010482 | 10 | 36417502 | 0.2193 | A/G | 5.53E-06 | 0.03798 |
| PCAT5,ANKRD30A | rs112089093 | 10 | 36411519 | 0.2201 | G/A | 5.88E-06 | 0.04153 |
| PCAT5,ANKRD30A | rs12248963 | 10 | 36401997 | 0.2032 | T/G | 1.97E-06 | 0.04222 |
| PCAT5,ANKRD30A | rs1480525 | 10 | 36398453 | 0.2032 | C/T | 1.97E-06 | 0.04248 |
| PCAT5,ANKRD30A | rs10128398 | 10 | 36414675 | 0.2201 | G/T | 5.88E-06 | 0.04348 |
| PCAT5,ANKRD30A | rs12256178 | 10 | 36416091 | 0.2201 | C/G | 5.88E-06 | 0.04395 |
| PCAT5,ANKRD30A | rs10128169 | 10 | 36412746 | 0.2193 | T/A | 5.53E-06 | 0.04726 |
| PCAT5,ANKRD30A | rs1122458 | 10 | 36394128 | 0.2002 | T/C | 1.02E-06 | 0.04839 |
| PCAT5,ANKRD30A | rs7071386 | 10 | 36413808 | 0.2193 | T/C | 5.53E-06 | 0.04863 |
| PCAT5,ANKRD30A | rs72793402 | 10 | 36415193 | 0.2201 | A/T | 5.88E-06 | 0.05475 |
| PCAT5,ANKRD30A | rs12264078 | 10 | 36395568 | 0.2178 | T/C | 5.31E-06 | 0.05513 |
| PCAT5,ANKRD30A | rs72793389 | 10 | 36408512 | 0.2193 | C/T | 5.53E-06 | 0.05589 |
| PCAT5,ANKRD30A | rs72793329 | 10 | 36407806 | 0.2201 | A/G | 5.88E-06 | 0.05961 |
| LOC101928283,GRM8 | rs4590382 | 7 | 125858717 | 0.04581 | A/G | 7.73E-06 | 0.05998 |
| PCAT5,ANKRD30A | rs11010458 | 10 | 36405364 | 0.2186 | T/C | 5.53E-06 | 0.06701 |
| PCAT5,ANKRD30A | rs7090573 | 10 | 36407380 | 0.2201 | C/T | 5.88E-06 | 0.06888 |
| PCAT5,ANKRD30A | rs16936700 | 10 | 36421152 | 0.2186 | A/G | 8.85E-06 | 0.07204 |
| PCAT5,ANKRD30A | rs7072062 | 10 | 36414157 | 0.2201 | T/G | 5.88E-06 | 0.07249 |
| PCAT5,ANKRD30A | rs11010438 | 10 | 36386254 | 0.2032 | T/G | 1.97E-06 | 0.07315 |
| PCAT5,ANKRD30A | rs12255912 | 10 | 36415683 | 0.2201 | A/G | 5.88E-06 | 0.07567 |
| PCAT5,ANKRD30A | rs11010469 | 10 | 36410747 | 0.2201 | G/A | 5.88E-06 | 0.07897 |
| PCAT5,ANKRD30A | rs72793316 | 10 | 36386383 | 0.2155 | G/A | 3.10E-06 | 0.08109 |
| PCAT5,ANKRD30A | rs72793330 | 10 | 36407826 | 0.2201 | A/G | 5.88E-06 | 0.09288 |
| PCAT5,ANKRD30A | rs10128151 | 10 | 36412580 | 0.2201 | C/A | 5.88E-06 | 0.09839 |

**Supplementary table 5 Putative regulatory function for top associated SNPs (P < 1.0E-8).**

| **Chromosome:**  **Location** | **dbSNP** | **Major Allele / Minor Allele** | **Gene** | **Position** | **Probability score of RegulomeDB** | **Histone marked** | **DNase** | **Motifs altered** | |
| --- | --- | --- | --- | --- | --- | --- | --- | --- | --- |
|  |  |  |  |  |  |  |  | Haploreg | RegulomeDB |
| 3:16407625 | rs139753473 | G/A | *RFTN1* | intronic | 0.00800 | + | + | E2F, Egr-1, MOVO, Nrf1, UF1H3BETA, YY1, SP1 | SP1, SP3 |
| 3:16407432 | rs200713609 | G/A | *RFTN1* | intronic | 0.60906 | + | + | PEBP |  |
| 3:16407472 | rs116358832 | A/G | *RFTN1* | intronic | 0.13454 | + | + | CEBPB, GATA |  |
| 3:16407446 | rs796950514 | T/C | *RFTN1* | intronic | 0.13454 |  | + |  |  |
| 3:16407458 | rs796881087 | A/G | *RFTN1* | intronic | 0.13454 |  | + |  |  |
| 3:16407456 | rs796687837 | A/G | *RFTN1* | intronic | 0.13454 |  | + |  |  |
| 3:16407448 | rs796068970 | A/G | *RFTN1* | intronic | 0.13454 |  | + |  |  |
| 3:16407440 | rs796689769 | C/T | *RFTN1* | intronic | 0.60906 |  | + |  |  |

Note: Expression quantitative trait loci (eQTL) data and bound proteins were not found for all of these SNPs in either Haploreg or RegulomeDB.
